# The MindSKILLZ sport-based mental health promotion intervention for adolescents in Kenya: a mixed methods pilot study

**DOI:** 10.3389/fpubh.2026.1746268

**Published:** 2026-03-02

**Authors:** Devyn E. Z. Lee, Bernard Nyauchi, Mercy Kihiu, Robert Kimathi, Elizabeth A. Okoth, Anthony Chazara, Christopher K. Barkley, Andrew Dallos, Stella Waruinge, Patrick Oyaro, Celina M. Kithinji, Ilan Cerna-Turoff, Ilana Cohen, Katherine G. Merrill, Lilian Otiso

**Affiliations:** 1 Grassroot Soccer, Inc., Hanover, NH, United States; 2 LVCT Health, Nairobi, Kenya; 3 Mental Health, Nairobi County Department of Health, Nairobi, Kenya; 4 Gender-Based Violence and Adolescents Health Program, Mombasa County Department of Health, Mombasa, Kenya; 5 Department of Epidemiology, Columbia University Mailman School of Public Health, New York, NY, United States; 6 Center for Dissemination and Implementation Science, University of Illinois Chicago, Chicago, IL, United States

**Keywords:** adolescent mental health and well-being, community-based mental health intervention, Grassroot Soccer, mental health promotion, sport-based intervention

## Abstract

**Background:**

Young people in Eastern and Southern Africa face critical mental health challenges, and most have little or no access to mental health care and support. MindSKILLZ is a sport-based mental health promotion and prevention intervention delivered to adolescents aged 10-14 by trained near-peer mentors (Coaches). A pragmatic pilot study was undertaken in Nairobi and Mombasa counties of Kenya to: (a) assess preliminary effects on adolescents' mental health outcomes (assets, depression, emotional conduct problems, and wellbeing); (b) explore effects on Coaches' mental health; and (c) understand program acceptability and potential sustainability and scalability.

**Methods:**

This pilot drew on mixed methods. An interrupted time series (ITS) design was used, with surveys administered to participants four times: twice before and twice after the intervention. Focus group discussions and key informant interviews were conducted with adolescent participants, Coaches, and key County Department of Health and implementing stakeholders post intervention. Data were analyzed using segmented fixed effects regression and a thematic qualitative analysis approach.

**Results:**

Surveys from 251 participants showed positive trends across all quantitative measures, though results were not statistically significant. Mental wellbeing, depression symptoms, mental health stigma, support seeking, emotional symptoms, and conduct problems improved from Time 2 (pre-intervention) to Time 3 (immediately post-intervention) – and these improvements mostly grew stronger at Time 4 (follow up). Participants described enhanced coping skills and improved stress and anger management from MindSKILLZ. Coaches described increased mental health knowledge, coping skills, patience, cooperation, and self-esteem. MindSKILLZ was highly acceptable, with key stakeholders mostly highlighting its potential for sustainability and scale given its low resource demands.

**Conclusions:**

Findings demonstrate high program acceptability and suggest promising effects on adolescent wellbeing and other key mental health outcomes. The intervention can potentially address a critical mental health service gap, as it can be universally delivered by lay providers.

## 1 Introduction

Mental health challenges are one of the most critical health issues facing young people today. Mental and substance use disorders are a leading cause of disability among young people, with an estimated 25% of adolescents experiencing depression and 20% experiencing anxiety, nearly double pre-COVID-19 levels (1, 2). While global adolescent mental health prevalence data is sparse, existing evidence suggests that the prevalence of common mental health disorders is highest in sub-Saharan Africa (SSA) (3). In Kenya, prevalence data for adolescent mental conditions is likewise scarce, but a recent nationally representative study found that over 44% of adolescents experienced a mental health problem in the past 12 months, with 12.1% meeting criteria for a mental health disorder (4).

Adolescence is a profound time of change and is a vital moment for establishing lifelong health habits. It is also a crucial developmental phase for mental health, with 50% of mental health conditions beginning by age 14, and 75% beginning by age 24 (5). Mental health is integral and essential to overall health outcomes. People with mental health challenges are between four and ten times higher risk of acquiring HIV, and depression is two to three times more common in people living with HIV (6).

Adolescents in Kenya face mental health challenges in multiple categories. As individuals, they may face a lack of parent/caregiver support, peer and social pressures, maladaptive coping mechanisms, drug and alcohol use, lack of awareness on mental health preventive measures, and a lack of autonomy over their own lives (7). The mental health service gap is great, as at most, only one out of every ten people with a mental health condition in Eastern and Southern Africa will ever receive professional care or treatment (8). Individuals who seek treatment face additional challenges at the level of service providers, including poor attitudes toward mental health issues, lack of confidentiality in healthcare settings, and limited promotive and preventive strategies to avert mental health issues (7).

In the context of high need and low resources to support adolescent mental health, group interventions delivered by trained lay providers are a promising approach to promote positive mental health in young people. School- and community-based interventions have strong potential to improve emotional and behavioral well being, as well as self-esteem, motivation, and self-efficacy (9). When these interventions are universally delivered—that is, not only delivered to adolescents with symptoms or mental health challenges—that can help avoid labeling individuals and increasing stigma. Universal delivery is also useful because even sub-clinical symptoms can cause distress and impairment (10). In sub-Saharan Africa, there is a clear gap in universally delivered mental health promotion interventions, as many focus on specific marginalized groups of young people, such as those living with HIV (11).

A global review of adolescent mental health program components found that interventions that addressed interpersonal skills, emotional regulation, and alcohol and drug education had significant effects across multiple outcomes (12). Programs that focus on the development of social and emotional learning show good evidence to build skills and reduce short-term depression and anxiety in young people in high-income countries. Additionally, the development of social and emotional skills can act as a protective factor for mental health challenges and a range of negative social, educational, and behavioral outcomes (13). Another approach shown to have positive effects on adolescents' mental health knowledge and awareness is that of interventions focusing on mental health literacy (13). Mental health literacy interventions aim to build understanding of how to obtain and maintain positive mental health, understand common mental health problems, decrease mental health stigma, and increase help-seeking self-efficacy (14).

While sport-based interventions have been used effectively in health promotion and education for several decades, sport-based programming for mental health promotion is still an emerging field (15). Participation in sport and physical activity provides numerous benefits for physical and mental health (16), and participating in group-based interventions encourages the formation of positive social connections, which also has positive effects on mental well being (17). Particularly in low-income settings, sport-based programming can act as an accessible, low-cost community-based intervention with great potential to improve youth mental health (17). Using non-competitive sport as a vehicle to engage young people and provide mental health information and skill-building offers a physical and emotional safe space for adolescents to develop their strengths to thrive (18).

Given the high burden of mental health challenges among adolescents in Kenya, limited access to mental health services, and need for universally delivered mental health promotion and primary prevention programming, Grassroot Soccer (GRS) and LVCT Health (LVCT) partnered with the County Governments of Mombasa and Nairobi to implement and study MindSKILLZ, a sport-based mental health promotion program for adolescents. The MindSKILLZ program, implemented in 12 sessions by trained, near-peer mentor ‘Coaches' from the community, aimed to improve mental health knowledge, skills, and attitudes, and decrease stigma around mental health.

In this study, we aimed to address the following objectives:

1. Assess the preliminary effects of MindSKILLZ on mental health outcomes (i.e., assets, depression, emotional conduct problems, and wellbeing) among adolescent participants.
2. Explore how facilitating MindSKILLZ influenced Coaches' own mental health.
3. Understand the acceptability and potential sustainability and scalability of the MindSKILLZ program in Kenya.

## 2 Materials and methods

### 2.1 Study design

The pilot study used mixed methods, with a pragmatic interrupted time series (ITS) design. Given the logistical challenges of enrolling a control group in this setting, an ITS design was selected as a rigorous quasi-experimental approach for examining intervention effects using a single population (19). Although the design was limited to four time points given feasibility constraints, this design was appropriate for a pilot study focused on examining preliminary signals of intervention effects and implementation outcomes. When combined with qualitative data, the ITS design provides valuable early evidence to inform a future, adequately powered trial (20, 21).

This study utilized quantitative survey data from adolescent MindSKILLZ participants to estimate the effect of the intervention on mental health and wellbeing. Qualitative data collected in focus group discussions with adolescent participants as well as young adult MindSKILLZ Coaches (i.e., program facilitators) was utilized to develop a deeper understanding of the experiences of intervention participants and implementers. Key informant interviews also were conducted with Department of Health personnel at the county level and with LVCT Health staff. To understand the program's acceptability and potential for sustainability, we drew on the implementation outcomes framework (22). We defined acceptability as the perception of stakeholders that the program was likable and satisfactory (22) and sustainability as the potential for the program to be continually delivered with adaptation as needed (23). Our goal was to collect initial data on acceptability and sustainability to inform future iterations and scale-up of MindSKILLZ. Acceptability is at the core of all implementation activities (24) and there is increasing recognition of the importance of planning for sustainability from a project's outset (25, 26).

### 2.2 Intervention description

MindSKILLZ utilizes Grassroot Soccer's sport- and play-based approach to learning about critical health topics. Grassroot Soccer has implemented sport-based health programming for youth since 2002 and utilizes a positive youth development approach that emphasizes fun and activity, relatable caring ‘Coach' mentors from the community, and evidence-based program content. Grassroot Soccer ‘SKILLZ' programming has shown effectiveness in improving adolescent health knowledge, building resilience and self-efficacy, and increasing uptake of health services (27–29).

The MindSKILLZ program was conceptualized by GRS, drawing guidance from key resources in adolescent mental health, such as the Inter-Agency Standing Committee (IASC) Guidelines, UNICEF's Helping Adolescents Thrive Toolkit, and relevant systematic reviews (9, 12). Key principles of trauma-informed mental health care were also integrated into MindSKILLZ, such as the importance of safety, trustworthiness, peer support, and empowerment (30).

The MindSKILLZ curriculum was then adapted and contextualized in a co-design workshop in Nairobi, Kenya in November 2022, a meeting that brought together members of the LVCT Health youth advisory committee, GRS and LVCT Health staff, representatives from County Departments of Health and the Ministry of Education. A total of 35 participants took part (21 female, 14 male) in activities across three days. Youth advisory committee members played an integral role in workshop activities, as participants discussed mental health challenges facing adolescents in Kenya, prioritized mental health topics to address in the program, developed a theory of change, and refined the program's activity framework. Engagement of government stakeholders ensured alignment with county and national level priorities for adolescent mental health. During the workshop, GRS and LVCT also discussed and agreed upon the duration and frequency of program sessions, based on experience delivering adolescent health programming. Input from Department of Health and Ministry of Education representatives, available time, resources, and ease of delivery were also considered in determining these decisions about program delivery during the workshop and in follow-up discussions.

The resulting MindSKILLZ program includes 12 sessions, covering topics including basic mental health knowledge, positive coping skills, identification of emotions, seeking care and support, identifying one's strengths, and standing up to peer pressure. A full list of session topics is included in Supplementary material, and Table 1 includes brief descriptions of several key MindSKILLZ activities.

**Table 1:** Description of illustrative MindSKILLZ activities.

**Activity name**	**Description**
Take five	Coaches guide participants through mindful, deep breathing, practicing inhaling and exhaling in different ways as a manner of focusing on breath. Key messages include that deep breathing can be helpful to calm down, focus energy, and help make good decisions.
Power hand	Participants trace one of their hands on paper, then think about their strengths and unique qualities and write one on each finger. They are encouraged to think of situations where it would be helpful to reflect on their strengths and things they are good at. Key messages include that everyone has positive attributes and reflecting on these can be a good coping skill.
Juggling my life	Participants stand in a circle and work as a team to keep a ball that represents a ‘healthy mind' in the air. They first pass the ball in a pattern, then the Coach adds additional balls that represent life stressors, making the activity more difficult. Key messages include that stress is a normal part of life, but too much is harmful, and we can develop skills to get better at managing stress.

A comic book-style print ‘magazine' was also developed to reinforce key content from in-person MindSKILLZ sessions (sample pages available in Supplementary material). GRS had used print magazines as a supplemental resource in previous programming, and team members in Kenya agreed it would be a useful addition. Program materials were field-tested with young people and translated into Swahili.

Coaches play a key role in the delivery of MindSKILLZ and other GRS programs, as they facilitate program sessions and act as positive, relatable role models for adolescent participants. As such, recruitment and training of Coaches was an important part of the intervention process. Coaches were recruited in Nairobi and Mombasa Counties by LVCT Health, with guidance from GRS. Positions were advertised via existing youth networks, such as the Ministry of Health's Youth Champions for Health Network, with candidates required to have a high school education, basic facilitation skills, and passion for working with youth and/or community development. Once hired, Coaches underwent a 5-day training to equip them with the necessary skills and knowledge to deliver MindSKILLZ: one training was held in Mombasa, and one was held in Nairobi, with Ministry of Health Officials present at both trainings.

In addition to providing key mental health information and skills of young people, the MindSKILLZ program also connected young people to additional mental health support beyond the program sessions. Coaches referred participants in need of additional support to the LVCT one2one platform (a youth hotline and digital health platform that includes chatbots, peer mentors, and professional counselors) or appropriate county health services.

**Figure 1:**
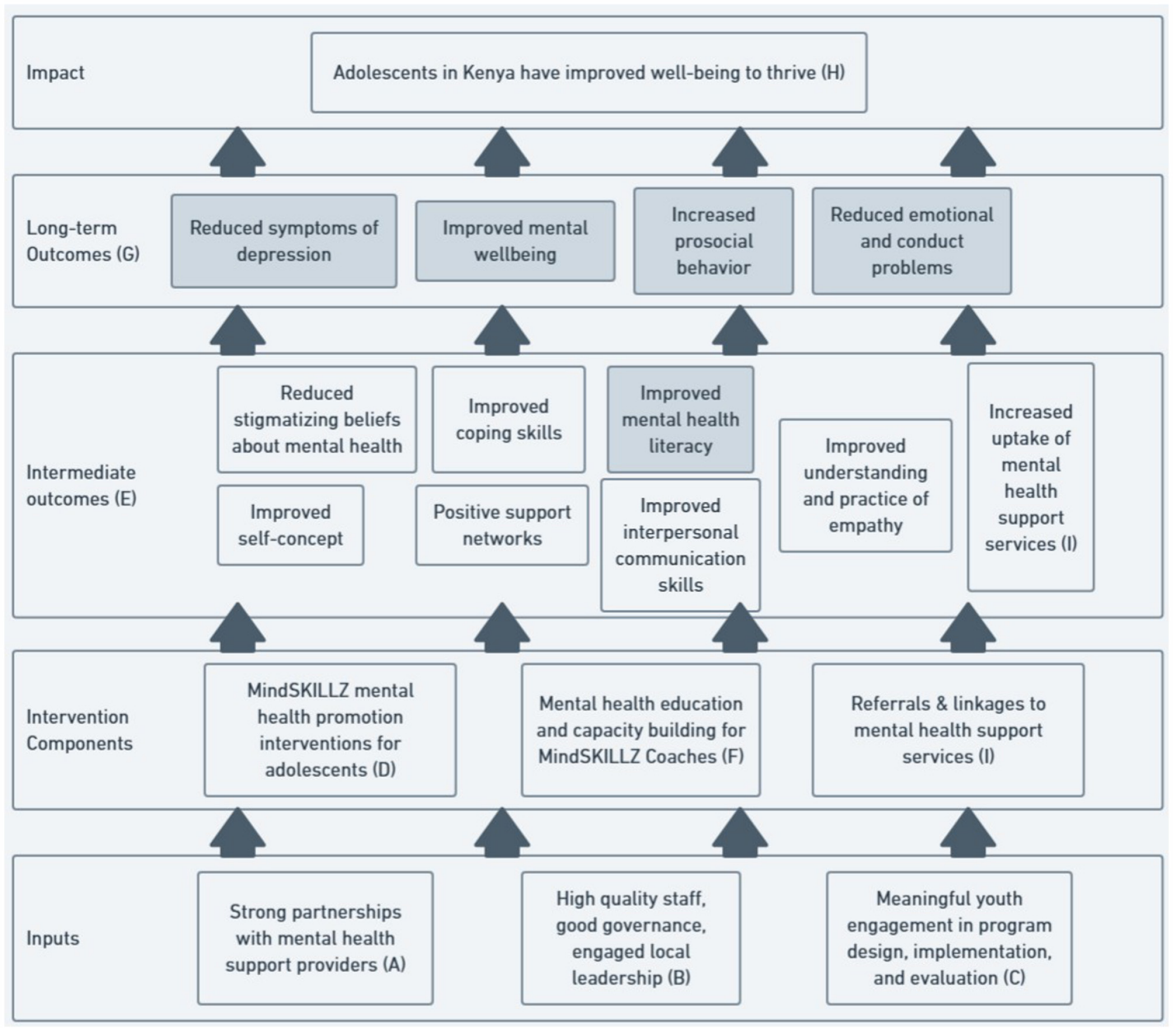
MindSKILLZ pilot study conceptual model.

The conceptual framework for the MindSKILLZ program in this pilot study is presented in Figure 1, illustrating how key program inputs and intervention activities are expected to lead to intended outcomes and ultimate impact. Key inputs for MindSKILLZ include engagement at the community level to form strong partnerships with mental health service providers (A), and engage local leadership (B). Meaningful youth engagement (C) across program design, implementation, and evaluation follows positive youth development principles central to GRS work. At the intervention components level, the MindSKILLZ program content (D) aims to improve multiple areas of knowledge, skills, and attitudes of youth, measured as intermediate outcomes (E), where the shaded outcome of mental health literacy is presented in this study. The mental health capacity building of Coaches (F) prepares them to deliver intervention sessions and develops them as positive role models and social support for adolescent participants. The longer-term outcomes measured by the study (G) are at the level of individual experiences of mental health challenges, which further input on the impact level of adolescent wellbeing to thrive (H). Additionally, referrals and linkages to mental health support services and their uptake (I), are considered intermediate outcomes. In this paper, we present the shaded intermediate and long-term outcomes with additional presentation of intermediate outcomes planned in future publications.

### 2.3 Participants and procedures

Data were collected in Mombasa and Nairobi Counties between August 2023 and March 2024. Quantitative and qualitative data collection were conducted by trained research assistants in sites purposively selected by LVCT through engagement with county and sub-county focal points to represent a variety of implementation settings (e.g., urban informal settlements, more affluent areas, etc.) and to include areas with high numbers of adolescents aged 10–14 years. In Nairobi County, the selected study sites were in Ruaraka and Westlands sub-Counties; participants were recruited in both school- and community-based settings, with Coaches engaging community health promoters and teachers for support. In Mombasa County, the selected study sites were in Likoni, Jomvu Changamwe, and Kisauni sub-counties; participants were recruited only in community-based settings. Inclusion criteria for adolescents were: aged 10–14 and enrolled in a MindSKILLZ intervention group in a selected study site. Within each selected study site, all participants in the selected intervention groups were invited to take part in the study. For MindSKILLZ Coaches, LVCT Health staff, and county health staff, the inclusion criteria were: involved in MindSKILLZ intervention planning or implementation and an adult (aged 18 years +).

Quantitative data were collected from adolescents. Research assistants administered the quantitative survey to participants at Time 1, and three to four weeks later at Time 2. Participants then proceeded through the 12-session MindSKILLZ program and were administered the Time 3 survey immediately after the intervention. The Time 4 survey was administered three to four weeks after Time 3. Across all time points, participants reported on measures of preliminary effectiveness. At Times 3 and 4, participants reported on measures of intervention acceptability.

Qualitative data were collected from all participant groups and included focus group discussions and key informant interviews. We conducted eight focus group discussions in total. Three focus groups with adolescent participants were held in Nairobi and two were held in Mombasa (*n* = 33 participants total). Two focus groups were held with Coaches in Nairobi and one was held in Mombasa (*n* = 23 total participants). We conducted two key informant interviews with County Health Department staff, one in Mombasa, and one in Nairobi, and three key informant interviews with LVCT Health staff (two in Nairobi and one in Mombasa). Adolescent focus group participants were purposively selected to represent both school- and community-based implementation groups, while key informant interview respondents were selected based on their involvement in MindSKILLZ program implementation and knowledge of the program. Data were collected post-intervention (i.e., after the Time 3 survey), and respondents received a transportation reimbursement for their travel to a central location.

Ethical approval for this study was obtained from Amref Health Africa's Ethics and Scientific Review Committee (ESRC-PP1389/2023). Adult participants provided written informed consent and adolescents (all minors under age 18) provided written informed assent with written parent permission.

### 2.4 Quantitative measures

The quantitative survey administered to adolescent participants drew on validated scales where possible and was pre-tested with adolescents prior to the first administration. Surveys addressed demographic characteristics (e.g., gender, age) and the following constructs.

Mental wellbeing: The WHO-5 is a brief, 5-item scale measuring mental wellbeing in the prior two weeks. It is among the most widely used scales for measuring psychological wellbeing, has been translated into over 30 languages, and has demonstrated acceptable psychometric properties, including among adolescents (31, 32). Scores range from 0-low wellbeing to 100-high wellbeing. A change in 10 points on the scale is considered clinically meaningful. Adolescents were classified as having greater levels of difficulty (i.e., poor wellbeing and possible depression) if they had scores of ≤ 50 (33).

#### 2.4.1 Depressive symptoms

The PHQ-9 is a 9-item, widely-used scale for measuring depressive symptoms over the past two weeks and has been validated among adolescents in Kenya (34, 35). Scores range from 0- no depressive symptoms to 27-high depressive symptoms with the following interpretations by score category: 0–4 – no depression symptoms, 5–9 – mild depression symptoms, 10–14 – moderate depression symptoms, 15–19 – moderately severe depression symptoms, and 20–27 – severe depression symptoms. A change in five points is considered clinically meaningful. Adolescents were classified as having greater levels of difficulty (i.e., moderate to severe depressive symptoms) if they had scores of ≥10 (35).

#### 2.4.2 Mental health literacy

We selected a subset of items from the Mental Health Literacy scale (MHLS) to examine self-efficacy for seeking mental health support (nine items) and stigmatizing beliefs around mental health (four items) (36). Scores for the support-seeking sub-scale ranged from 4-low support seeking to 20-high support seeking and for the stigma sub-scale from 5-low stigma to 45-high stigma.

#### 2.4.3 Psychological attributes

The Strengths and Difficulties Questionnaire (SDQ) is a brief behavioral screening questionnaire that assesses multiple dimensions of mental health (37). We selected the emotional symptoms, prosocial behavior, and conduct problems sub-scales (five items each, 15 items total) as they were most relevant to MindSKILLZ. Each sub-scale ranged from 0–10. For the emotional symptoms and conduct problems sub-scales, higher scores indicate higher levels of difficulty, whereas for the prosocial behavior sub-scale, higher scores indicate lower levels of difficulty. Adolescents were classified as having greater levels of difficulty if they had scores of ≥5 on the emotional symptoms or conduct problems sub-scales (i.e., slightly raised to very high) or ≤ 6 on the prosocial behavior sub-scale (i.e., slightly lowered to very low) (37).

#### 2.4.4 MindSKILLZ intervention acceptability

We drew on seven items from the Mental Health Implementation Science Tools (mhIST) acceptability measure, which has shown strong psychometric properties (38). Scores per item range from 0-low acceptability to 3-high acceptability.

### 2.5 Qualitative tools

Focus group discussions used a semi-structured guide, developed by the research team and field-tested with adolescents. The guide for adolescents included questions on program content and activities, likes and dislikes, recommendations for program improvement, and reported changes in behavior, knowledge, and attitudes around mental health. For example, ‘What are some of the things that you learned in MindSKILLZ sessions,' and ‘Can you give any examples of how mental health challenges can impact peoples' lives?' The guide for Coaches included questions on their views on program content and activities, their observations of changes in participants, and recommendations for improving MindSKILLZ implementation. For example, ‘How well do you think participants learned about mental health and wellbeing in MindSKILLZ,' and ‘What did you personally learn about mental health, if anything, through your work as a Coach?' The key informant interview guide with County Health Department and LVCT Health staff included questions about program acceptability and potential sustainability and scalability. For example, ‘How resource-intensive is MindSKILLZ, compared to other programs addressing the same needs?'

### 2.6 Analyses

For quantitative data, descriptive statistics were used to summarize and tabulate data at each time point. To assess preliminary intervention effects on the outcomes of interest, we used piecewise or ‘segmented' fixed effects linear regression, a common method for interrupted time series (ITS) designs. The introduction of the MindSKILLZ intervention immediately following Time 2 (out of four time points) served as the “interruption,” creating a pre- intervention and post-intervention segment. We assessed whether there was an immediate effect of MindSKILLZ (i.e., a change in outcome level) by comparing the period prior to its introduction (i.e., from Time 1 to Time 2) with the period following implementation (i.e., from Time 2 to Time 4).

We ran fixed effects segmented regression models for each outcome in the full sample and then stratified analyses by sex, site, and age. For mental wellbeing, depression, and strengths and difficulties, we examined intervention-related changes among participants reporting greater difficulties at baseline. Specifically, we examined the proportion of participants reporting greater difficulties across the four time points and calculated the percent change between Time 2 (immediately before) and Time 3 (immediately after) the intervention, statistically compared the difference using a z test. We then used fixed effects segmented regression to examine changes following the intervention among those reporting greater difficulties at Time 1.

For all fixed effects regression models, we checked for autocorrelation for each model, and where autocorrelation was present, we accounted for it using robust estimates of variance. These analytic approaches were selected to minimize bias from stable confounders and underlying temporal trends, while acknowledging the exploratory nature of this pilot study.

For qualitative data, trained research assistants transcribed all recorded focus group discussions and interviews and translated them from Kiswahili to English where needed. Independent reviewers verified the quality of translated transcripts. Using NVivo, two research assistants coded the qualitative data, first developing a coding framework, then using a combined inductive and deductive approach and creating themes based on the framework (39). Themes were presented to the larger study team and agreed upon through discussion.

## 3 Results

### 3.1 Sample characteristics

Surveys at all four time points were completed by 256 participants. Five participants whose dates of completion were not consecutive were dropped, leaving 251 participants in the final sample. Just over half of participants were female (56.2%). Participant age was relatively evenly distributed from 10 years to 14 years, with the greatest proportion of participants aged 10 years (24.3%). Most participants were in grades 4–6 at school (63.4%), followed by grades 7–9 (25.1%), then grades 1–3 (11.6%) (Table 2).

**Table 2:** Demographic information of adolescent study participants (*n* = 251).

**Characteristic**	***n* (%)**
**Gender**	
Male	110 (43.8%)
Female	141 (56.2%)
**Site**	
Mombasa	130 (51.8%)
Nairobi	121 (48.2%)
**Age**	
10 years	61 (24.3%)
11 years	47 (18.7%)
12 years	58 (23.1%)
13 years	59 (23.5%)
14 years	26 (10.4%)
**Grade**	
Grades 1–3	29 (11.6%)
Grades 4–6	159 (63.4%)
Grades 7–9	63 (25.1%)

### 3.2 Quantitative results on preliminary effects of MindSKILLZ

Across all measures, scores trended in the desired direction but did not reach statistical significance (Table 3). Mental wellbeing, depression, mental health stigma, support seeking, emotional symptoms, and conduct problems all moved in the desired direction from Time 2 (pre-intervention) to Time 3 (~9–12 weeks post-intervention). No notable differences were observed by gender or age. Importantly, desired changes showed signs of persisting and growing stronger for mental wellbeing, depression, and mental health support-seeking based on the mean scores at Time 4.

**Table 3:** Mean scores for measures over time (*n* = 251 participants).

**Outcomes and measurement tools**	***n***	**Pre-intervention**		**Post-intervention**		***p* value**
		**Time 1**	**Time 2**	**Time 3**	**Time 4**	
Mental wellbeing (WHO-5)	251	74.1	74.4	76.2	80.0	0.51
Depressive symptoms (PHQ-9)	237	5.4	5.1	4.4	3.7	0.59
**Mental health literacy (MHLS)**					
Mental health stigma	251	25.3	24.8	22.9	22.5	0.19
Mental health support seeking	251	14.5	14.7	16.1	16.7	0.34
**Strengths and difficulties (SDQ)**					
Emotional processing difficulty	229	4.8	4.5	4	4.1	0.17
Conduct problems	232	3.2	3.2	2.9	2.9	0.41
Prosocial behavior	234	8.6	8.5	8.4	8.7	0.16

*p*-values compare slope of change between time 1–2 (pre intervention) to time 2–4 (post-intervention) using fixed effects regression models.

**Figure 2:**
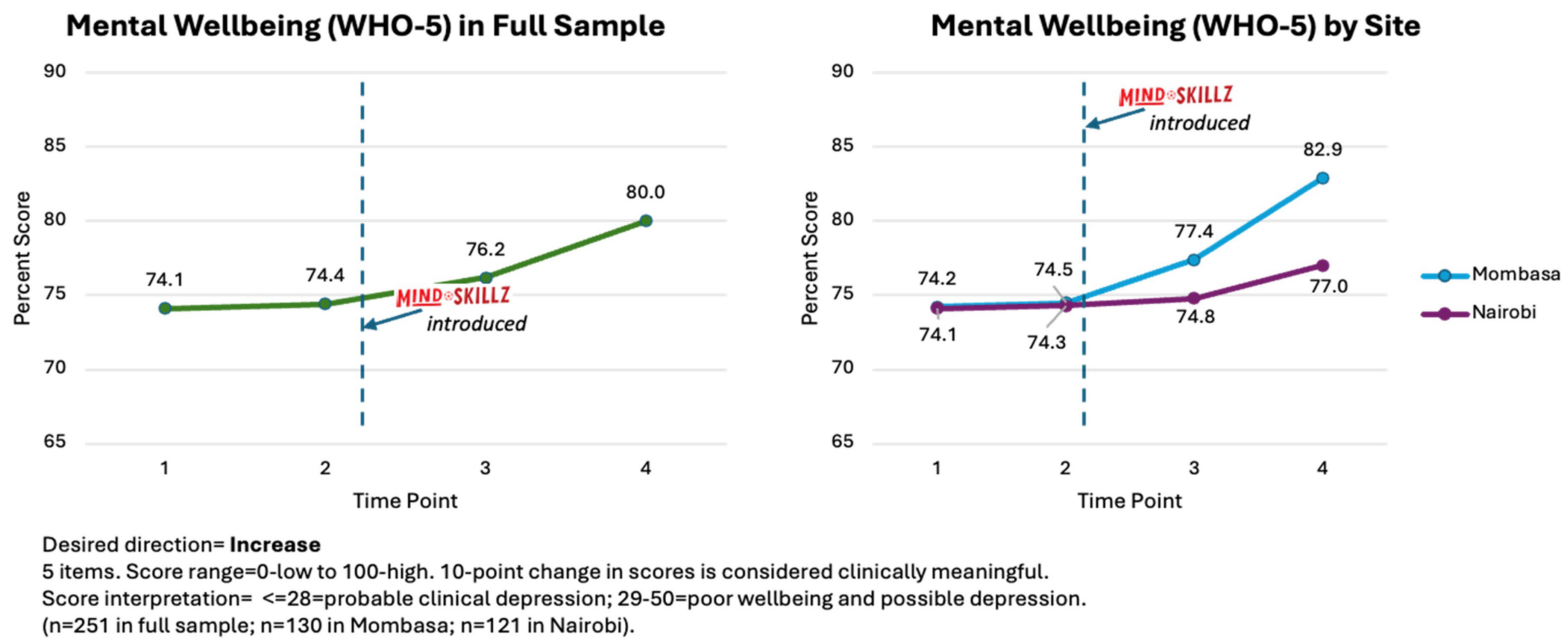
Changes in mean scores for mental wellbeing (WHO-5) across time in the full sample and by site (Mombasa vs. Nairobi).

For mental wellbeing, average scores on the WHO-5 in the full sample ranged from 74.1 (Time 1) to 80.0 (Time 4). These scores are in the range representing the presence of mental wellbeing (i.e., 51–100). When examining average scores separately by site, participants in Mombasa appeared to show greater improvement in WHO-5 scores post-intervention compared to those in Nairobi, but these differences did not reach statistical significance (Figure 2).

**Figure 3:**
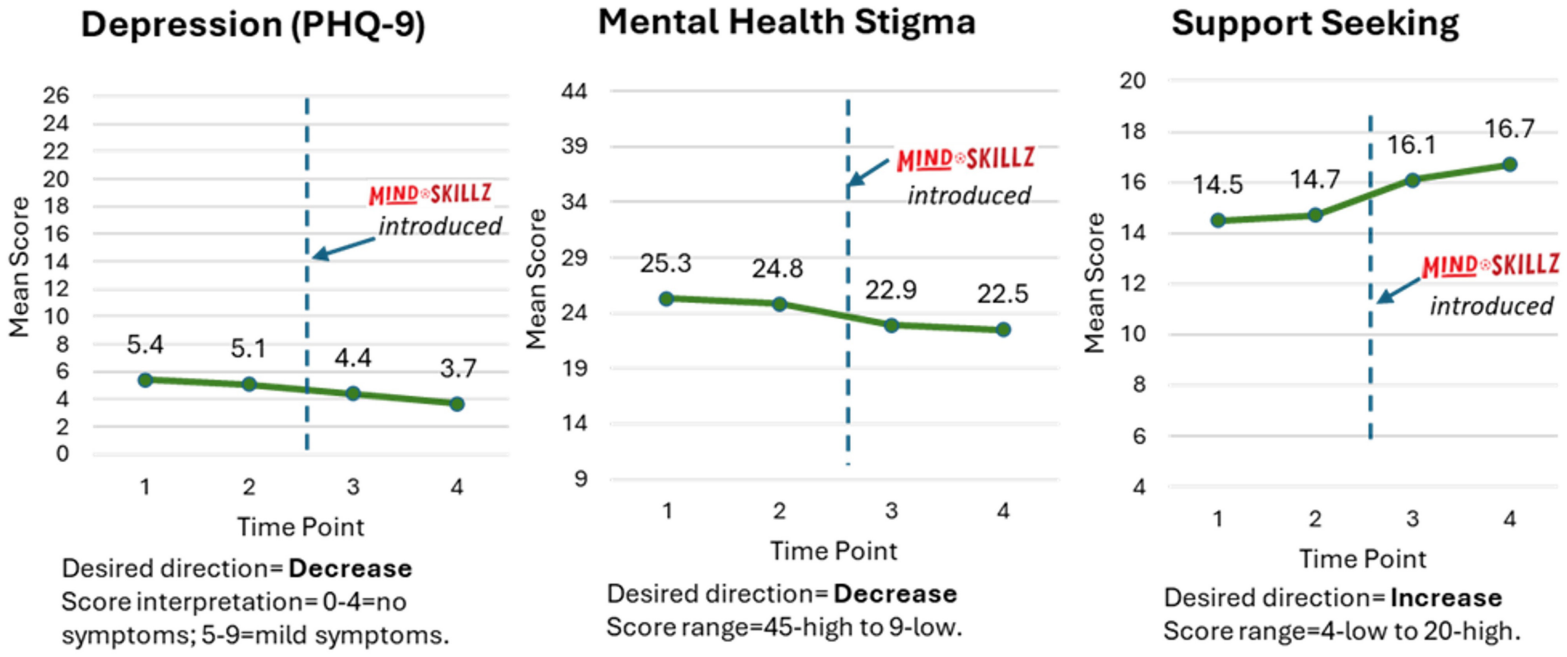
Changes in mean scores for depression and mental health literacy (stigma and support-seeking) across time in the full sample. MindSKILLZ logo used with permission from Grassroot Soccer, Inc.

For the depression measure (PHQ-9), average scores in the full sample pre-intervention were in the ‘mild depressive symptoms' range (i.e., 5–9 points). These scores decreased to the ‘no depressive symptoms' range (i.e., 0–4 points) post-intervention (scores of 4.4 at Time 3 and 3.7 at Time 4 on a range of 0–4). On the Mental Health Literacy Scale, average scores were generally in the middle of the response range for mental health stigma (between 22 and 25 on a scale of 5 to 45) and toward the upper end of the scale for support seeking (between 14.5 and 16.7 on a scale from 4 to 20). Each scale showed improvements of a few points, respectively, before and after the intervention. These differences were not statistically significant (Figure 3).

On the Strengths and Difficulties Questionnaire (Table 4), in the full sample, participants were in the ‘close to average' range for emotional symptoms (scores of 4 on a range of 0–4), conduct problems (scores of 2 to 3 on a range of 0–4), and prosocial behavior (scores of 8 on a range of 7–10). Score changes from pre- to post-intervention were small but in the desired direction.

**Table 4:** Proportions of participants reporting mental health difficulties over time.

**Sample**	Pre-intervention		Post-intervention		From time 2 to time 3	
	**Time 1**	**Time 2**	**Time 3**	**Time 4**	**Percent change**	*p* value^*^
WHO-5 moderate/severe symptoms (< = 50)	23 (9.2%)	39 (15.5%)	21 (8.4%)	20 (8.0%)	46% decrease	0.44
PHQ-9 moderate/severe symptoms (10 + )	41 (17.3%)	40 (16.9%)	33 (13.9%)	22 (9.3%)	17% decrease	0.72
SDQ emotional processing difficulty (5 + )	125 (54.6%)	111 (48.5%)	88 (38.4%)	93 (40.6%)	21% decrease	0.15
SDQ conduct problems difficulty (5 + )	51 (22.0%)	52 (22.4%)	45 (19.4%)	48 (20.7%)	13% decrease	0.72
SDQ prosocial behavior difficulty (< 7)	32 (13.7%)	32 (13.7%)	39 (16.7%)	31 (13.3%)	22% increase	0.73

^*^Compares the proportion at time 2 and time 3 using a *z* test.

The proportion of participants reporting mental health difficulties on the WHO-5, PHQ-9, and SDQ declined from pre-intervention (Times 1 and 2) to post intervention (Times 3 and 4) with the exception of prosocial behavior difficulty, though differences from Time 2 to Time 3 did not reach statistical significance (Table 4). On the WHO-5, we observed a 46% decrease in the proportion of participants showing poor mental wellbeing and possible depression from Time 2 (immediately pre-intervention) to Time 3 (immediately post-intervention).

**Figure 4:**
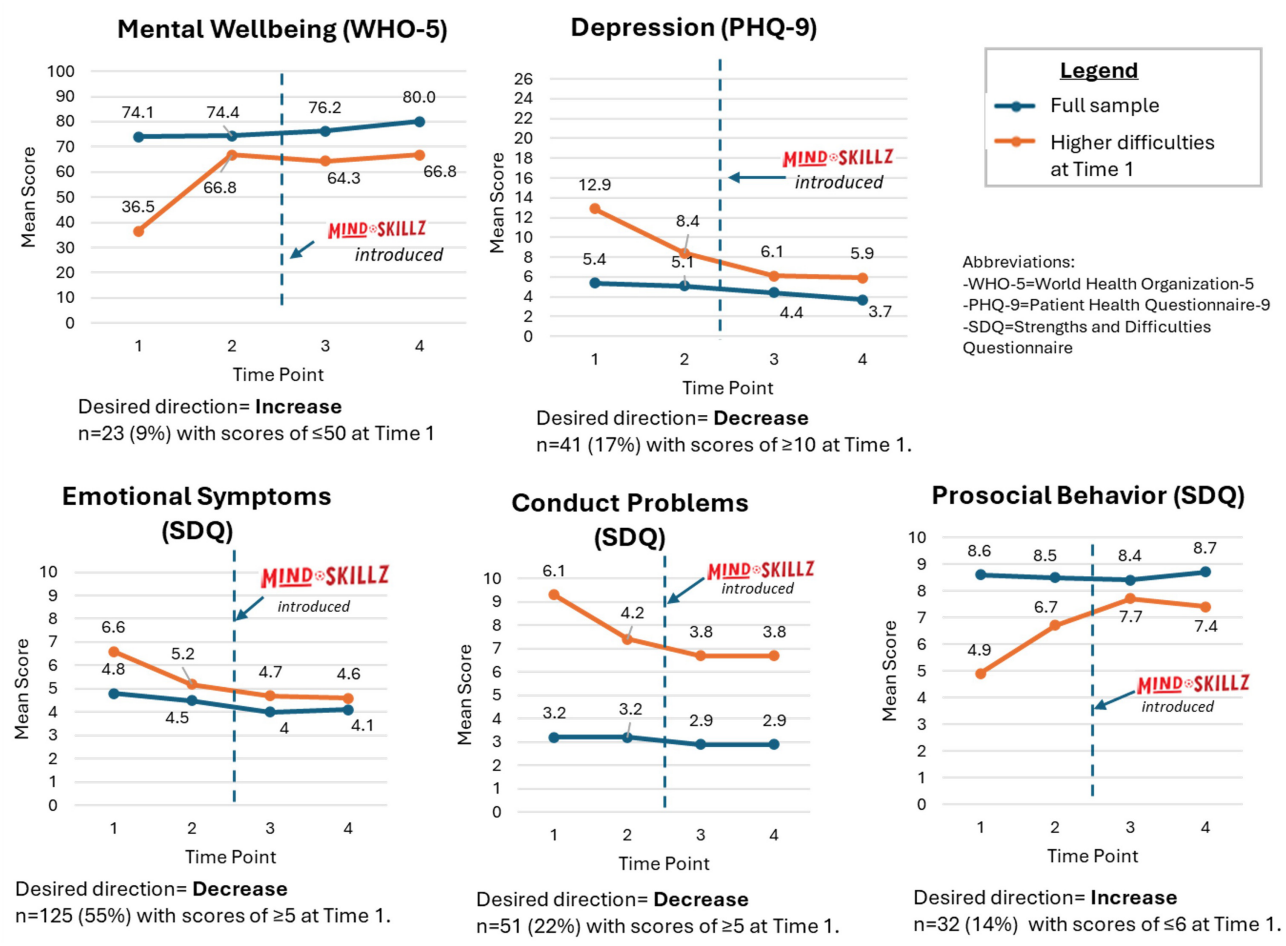
Changes in mean scores over time for participants showing greater difficulties at Time 1 compared to the full sample of participants. MindSKILLZ logo used with permission from Grassroot Soccer, Inc.

Participants showing mental health difficulties at Time 1 suggest greater changes in their scores for mental wellbeing, depression, and strengths and difficulties between Time 1 and Time 4 compared to the full sample of participants (Figure 4). However, interestingly, for each of these measures, the most notable jumps in these scores occurred from Time 1 to Time 2, prior to the introduction of MindSKILLZ. Some of these changes between Times 1 and 2 were clinically significant (e.g., for mental wellbeing and depression).

### 3.3 Qualitative findings on preliminary effects of MindSKILLZ

MindSKILLZ participants described enhanced coping skills and improved management of anger and stress, changes that were also observed and conveyed by Coaches and key stakeholders. In focus group discussions, adolescent participants described learning through MindSKILLZ about mental health concepts, increasing their understanding and changing their attitudes about the meaning of mental health:

“*We learned about mental illnesses, and we are told mental illnesses are common. It's not just going crazy. Some of them is being anxious, being stressed, depression. It's not just going mad.”* (Adolescent focus group, Nairobi)

Adolescent respondents accurately explained the concept of mental health when prompted and provided accurate examples of common mental health challenges. Adolescents were also asked how they would advise a friend experiencing mental health challenges, and most responded that they would encourage their friend to join MindSKILLZ. If the friend would not join, many adolescents explained that they would teach their friend about what they had learned in MindSKILLZ, particularly the ‘Take 5' mindful breathing exercise. They also provided multiple examples of where a friend could seek help, such as by calling 1190 (LVCT's one2one hotline) or going to a hospital, in addition to consulting with MindSKILLZ Coaches.

MindSKILLZ participants explained that the program improved their ability to recognize and make sense of their own emotions as well as those of other people. The program helped them understand their actions and reactions to certain situations, as well as those of peers. Adolescents in both Nairobi and Mombasa Counties described valuable skills they gained from MindSKILLZ, including skills in anger and stress management and communication:

“*In MindSKILLZ, I learnt how to breathe in, and breathe out…. to get rid of anger*.” (Adolescent focus group, Mombasa)

Adolescents reported feeling more confident in coping with stress and handling situations they were previously afraid of. The use of breathing exercises and the “power hand” technique were particularly popular skills that had a positive impact on the adolescents:

“*The time that we started, sometimes other friends could be angry about something that is just normal to happen like a mistake, but now they usually control their temper and right now when you do a mistake, they can forgive you.”* (Adolescent focus group, Nairobi)

MindSKILLZ Coaches and key stakeholders echoed observing positive changes in adolescent participants, providing numerous examples from their intervention groups. For example:

“*Kids were like not really aware of ‘What are we going to do?' Especially when we tell them, ‘It's mental health and we are going to use games to administer this toolkit.' And they would be like, ‘Wow! Mental health- this is a story about mad people.' But again, as we move, you find that they understand what stress really is, and even if maybe the other person or maybe the other kid has stress, now they can be able to understand.”* (Coach focus group, Mombasa)

### 3.4 Qualitative findings on MindSKILLZ Coaches' own mental health

MindSKILLZ Coaches in both Nairobi and Mombasa described how MindSKILLZ had a positive impact on them as individuals. They explained that it helped build their mental health knowledge and provided them with practical skills in managing anger and stress. For example:

“*I knew about mental health before we started, but I think with MindSKILLZ, it was more elaborated. It was more practical. I only knew what is mental health and what [it is] not, but in the terms of the coping skills, I learned them through MindSKILLZ …Personally I have an issue with anger a lot. I am that person who, if something happens, my anger rises up from 10 to 150 [degrees] onwards. [Now] I know if something happens, if I'm in a lot of pressure, I use the “Take Five” [strategy] almost every day.”* (Coach focus group, Mombasa)

One Coach in Nairobi also explained that their MindSKILLZ training helped them support a family member experiencing a mental health challenge. The Coach described learning about where to seek help and viewing the mental health problem in a similar light to a physical health problem, that there was no shame in seeking care.

Coaches highlighted that MindSKILLZ helped them manage strong emotions and resolve conflicts with others in a healthy manner. For example:

“*MindSKILLZ has impacted me as an individual. At first, I used to be judgmental and then when one maybe could wrong me at first, I used to judge them or start ranting without knowing the cause…. After going through the MindSKILLZ program, I now understand maybe someone can do something and it's not because they want to do it, but it's because even themselves they're facing a certain challenge. When I understand that aspect… when one wrongs me, I just looked at them, I don't rant anymore. I just smile and then later on when the person is settled, I come and find out maybe there is also a challenge that person is undergoing. No wonder the person maybe is having anger issues. Yeah, so nowadays I'm not judgmental. I just understand them [the other person] and deal with them the way they are and sometimes I also help them deal with their mental health issues.”* (Coach focus group, Mombasa)

Coaches also described how their role in MindSKILLZ helped them develop more patience, and a greater appreciation for cooperation and partnership through working with their coaching partners:

“*[Being a MindSKILLZ Coach] has really taught me patience, because dealing with kids 10 to 11 years...”* (Coach focus group, Nairobi)

“*To talk on that issue of partnership, working together…For you to deliver, you need to keep in mind that you have a partner. At all times, even if you're going for MindSKILLZ, we are different and we have different personalities. So, you get also to learn, ‘It's okay. I like doing my things this way, but my partner likes doing his things this way.' So how we come together and accommodate each other.”* (Coach focus group, Nairobi)

### 3.5 Program acceptability and potential for sustainability and scalability

MindSKILLZ was highly acceptable to participants, reflected in both the quantitative and qualitative results. On quantitative surveys, mean scores were 2.7 (standard deviation = 0.7) on the scale from 0-low to 3-high. An overwhelming majority of adolescents reported that they liked MindSKILLZ ‘a lot' (91%) and that they liked attending the sessions ‘a lot' (91%) (Table 3). Participants also reported that they found the skills they learned in MindSKILLZ to be useful (86% reporting ‘a lot'). Participants felt very comfortable raising questions to their MindSKILLZ Coach (79% reporting ‘a lot') and a large majority said that they understood the way things were explained (81% reporting ‘a lot') (Table 5).

**Table 5:** Acceptability ratings of the MindSKILLZ intervention.

**Acceptability item**	***n***	**Not at all**	**A little bit**	**A moderate amount**	**A lot**	**Mean (SD) (0-low to 3-high)**
Overall, did you like MindSKILLZ?	251	5 (2%)	10 (4%)	7 (3%)	229 (91%)	2.8 (0.6)
Did you like attending the MindSKILLZ sessions?	251	6 (2%)	2 (1%)	16 (6%)	227 (90%)	2.8 (0.5)
Did you like the MindSKILLZ magazine?	247	32 (13%)	2 (1%)	10 (4%)	203 (82%)	2.6 (1.0)
Did you feel that the skills you learned in MindSKILLZ are useful?	251	4 (2%)	7 (3%)	13 (5%)	227 (90%)	2.8 (0.5)
Did you feel comfortable raising questions to your MindSKILLZ Coach?	251	9 (4%)	21 (8%)	25 (10%)	196 (78%)	2.6 (0.8)
Did you feel that you understood the way things were explained to you during MindSKILLZ?	251	5 (2%)	11 (4%)	21 (8%)	214 (85%)	2.8 (0.6)
Did you feel that you understood the content of the MindSKILLZ magazine?	245	26 (11%)	15 (6%)	27 (11%)	177 (72%)	2.4 (1.0)
Total	251	12 (5%)	10 (4%)	17 (7%)	210 (84%)	2.7 (0.7)

Items are adapted from the mhIST (reference). Figures are *n* (%) or mean (standard deviation, SD). Coding is not at all = 0, a little bit = 1, a moderate amount = 2, and a lot = 3.

Qualitative findings revealed that the activities in MindSKILLZ were highly acceptable to adolescents, which they found engaging and interactive. Coaches, LVCT staff, and County Health staff observed that the play-based learning medium benefited participants. Adolescent participants reported that the games they played with their peers were a lot of fun, and recalled their favorite activities vividly:

“*My favorite section was the game we played with three balls. You throw and you pass the balls to another person and you look for the person whom you are expecting the ball from [Juggling My Life].”* (Adolescent focus group, Nairobi)

Participants provided positive feedback on the program, including use of near-peer mentor MindSKILLZ Coaches:

“*I really thank this mental health program. It really helped us a lot. And the Coaches were really good. They helped us cope with our stress really well.”* (Adolescent focus group, Nairobi)

County health stakeholders familiar with the program highlighted how much participants seemed to enjoy MindSKILLZ:

“*The session that I‘ve been able to attend I've interacted with the young ones, I‘ve seen them really enjoy the way the program is designed in the first place, the way the program is assisting them in … you know when they're coming together and having the games that they are playing together. But at the end of every game, there's a lesson learned.”* (County Health key informant interview, Mombasa)

Most LVCT and County Health staff felt that MindSKILLZ had the potential to be sustained and scaled. They described how MindSKILLZ required minimal resources for implementation, which can help facilitate its adoption and integration into various settings. These respondents emphasized that only two Coaches were needed to facilitate MindSKILLZ Program classes comprising a group of 25 children, which was considered a good Coach-participant ratio. This efficient resource utilization, according to the respondents, was useful in allowing for the enrolment of more participants without necessarily incurring additional costs. For instance:

“*The Government can sustain the program…. because it doesn't require a lot of resources…If one Coach or two Coaches can take up to 15 kids, you see that's a good number…There is a good ratio and more people can be enrolled in the program. So even in terms of the cost… it's one of the best models that we have actually.”* (County Health key informant interview, Mombasa)

Contrasting perspectives emerged about the program's potential sustainability and scalability from a few Coaches and one LVCT staff member in Nairobi. One Coach highlighted the unexpected cost of paying for intervention venues in areas where they had not yet established rapport with community leaders, who often later gave venue space at no cost. The LVCT staff member highlighted how additional resources (i.e., additional funds) were allocated for the pilot, noting that scaling the program could be costly:

“*We have found that we have done a lot of further resource allocation outside the program to support the entire program…. I think for us, when it comes to the scale, it's to get the results that we're getting, there's a lot of resource needed…”* (LVCT staff key informant interview, Nairobi)

## 4 Discussion

MindSKILLZ shows promise as a universally delivered, lay provider-facilitated intervention that fills critical gaps in mental health promotion and primary prevention programming for adolescents in Kenya (11, 40). All quantitative mental health measures trended in the desired direction, though not reaching statistical significance given a low study power in this pilot. MindSKILLZ participants described improved coping skills and management of anger and stress, changes which were also observed and noted by Coaches and key stakeholders. Participants also reported improved mental health knowledge, including where to access services and how to support friends. Coaches described how their role in MindSKILLZ helped them build their mental health knowledge and coping skills, as well as supporting them to improve their patience, cooperation, and self-esteem. The program was highly acceptable to participants, and key stakeholders viewed the program positively, with good potential for sustainability and scalability.

A key finding from this study was that positive trends in quantitative outcomes were observed to continue from Time 3 to Time 4 after the completion of the intervention. Studies have found that intervention effects often diminish over time according to the *fadeout* phenomenon (41). The apparent absence of fadeout effects may suggest that MindSKILLZ participants have gained skills to cope with challenges and lessen their experiences of poor wellbeing and other mental health symptoms over time. Future studies of MindSKILLZ should further explore these trends from the pilot with a larger sample size and measurement of at multiple time points to understand potential changes over time.

While MindSKILLZ is designed to be universally delivered to all adolescents, the intervention may especially support adolescents experiencing mental health difficulties. The proportions of adolescents reporting mental health difficulties declined following the intervention, with the most notable decrease in poor wellbeing and possible depression as measured by the WHO-5. Subgroup analyses among those with worse mental health at Time 1 showed some clinically significant changes, but these occurred between Time 1 and Time 2—that is, *before* participants were exposed to MindSKILLZ programming. It is possible that simply having the space to report their challenges on a survey may have lessened symptoms experienced by some participants. This phenomenon has been observed in other studies, where the largest change in reported depression and anxiety symptoms was observed after the first survey of several repeated measures but before changes were expected (42). The effects of MindSKILLZ on youth experiencing greater mental health challenges at baseline should be assessed in a future fully powered study.

MindSKILLZ was highly acceptable, demonstrated by both quantitative and qualitative data: nearly all participants reported that they enjoyed the program and liked attending the sessions. The engaging, interactive activities were highly enjoyed by participants, and adult respondents highlighted that the play-based learning medium benefited participants. The inclusion and meaningful engagement of youth advisory committee members in the design process likely contributed to high program acceptability, as early inclusion of adolescents in programs designed to benefit them has been found to positively affect program acceptability (43). Acceptability is necessary for high uptake and effectiveness of interventions, and the high acceptability of MindSKILLZ is important to its success and potential scale (44). While findings on sustainability and scale were mostly positive, GRS and LVCT are responding to concerns raised about the costs of the program by exploring what a ‘minimum package' for MindSKILLZ would look like in a future study. Ongoing work includes the development and evaluation of adapted delivery models with fewer in-person sessions, as well as calculation of the costs of longer vs. shorter interventions.

Study limitations should be noted. Given the small number of time points and the limited sample size, our study was likely underpowered, which could explain the lack of statistically significant findings despite all measures trending in the desired direction. The ITS design used only four time points (two on either side of the intervention delivery), whereas segmented regression typically requires at least eight to twelve data points on other side of the intervention delivery for validity (45, 46). Having only two data points before the introduction of MindSKILLZ makes it challenging to establish a trend to serve as a true comparison while also constraining our ability to adjust for time-varying confounders, such as concurrent contextual changes. Additionally, data were collected over four months total, whereas at least a full calendar year is recommended for ITS designs to account for possible biases introduced by seasonality– though we do not expect seasonality to have played a notable role in this study. Qualitative data collection with participants was done between Times 3 and 4, which could have affected quantitative responses at these time points, but qualitative data collection was conducted with a small proportion of the sample thus is unlikely to have affected the findings.

## 5 Conclusions

This pilot study of the MindSKILLZ program shows promising results, including signals of effects on key mental health outcomes for adolescent participants. Both adolescents and Coaches described improvements to their mental health through MindSKILLZ, including improved mental health knowledge and coping skills, as well as improved patience, cooperation, and self-esteem. MindSKILLZ was highly acceptable among adolescents, who appreciated the play-based methodology. Key stakeholders also viewed the program positively and noted its potential sustainability and scale. Early findings suggest that MindSKILLZ may fill a programming gap and address documented needs in the mental health promotion and prevention space. These pilot findings demonstrate the feasibility of recruiting and retaining participants and collecting repeated outcomes data and will inform the design of a fully powered controlled trial to rigorously assess the effectiveness and implementation of MindSKILLZ.

## Data Availability

The raw data supporting the conclusions of this article will be made available by the authors, without undue reservation.
