# Supplementary material for: The MindSKILLZ sport-based mental health promotion intervention for adolescents in Kenya: a mixed methods pilot study
Source: Front Public Health. 2026 Mar 2;14:1746268. doi: 10.3389/fpubh.2026.1746268 (PMC12990851; doi:10.3389/fpubh.2026.1746268)
Supplement: Supplementary file 1 [file Table_1.docx]

Supplementary Material

# Supplementary Figures

**Supplementary Figure 1: Overview of MindSKILLZ Session Topics**


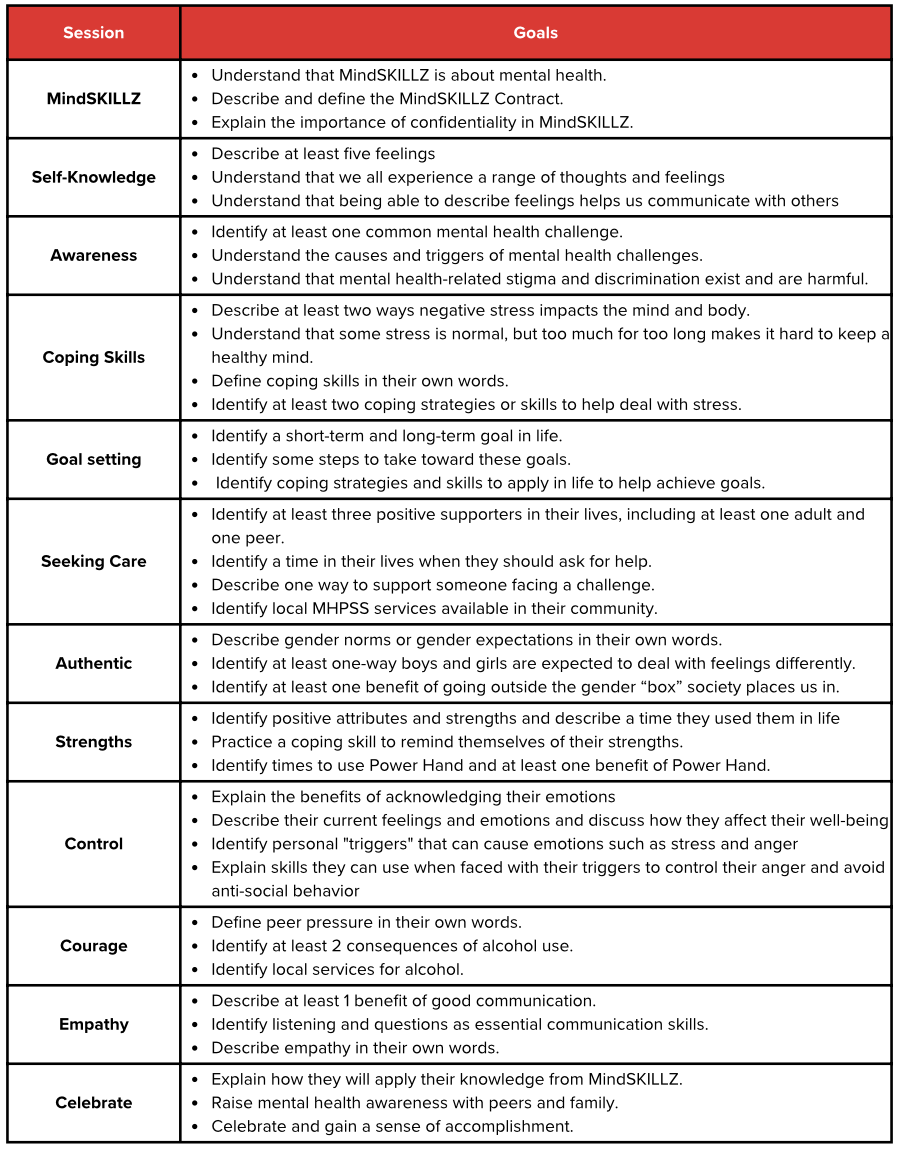


**Supplementary Figure 2: Sample pages from MindSKILLZ Magazine. Reprinted with permission from Grassroot Soccer, Inc.**

**
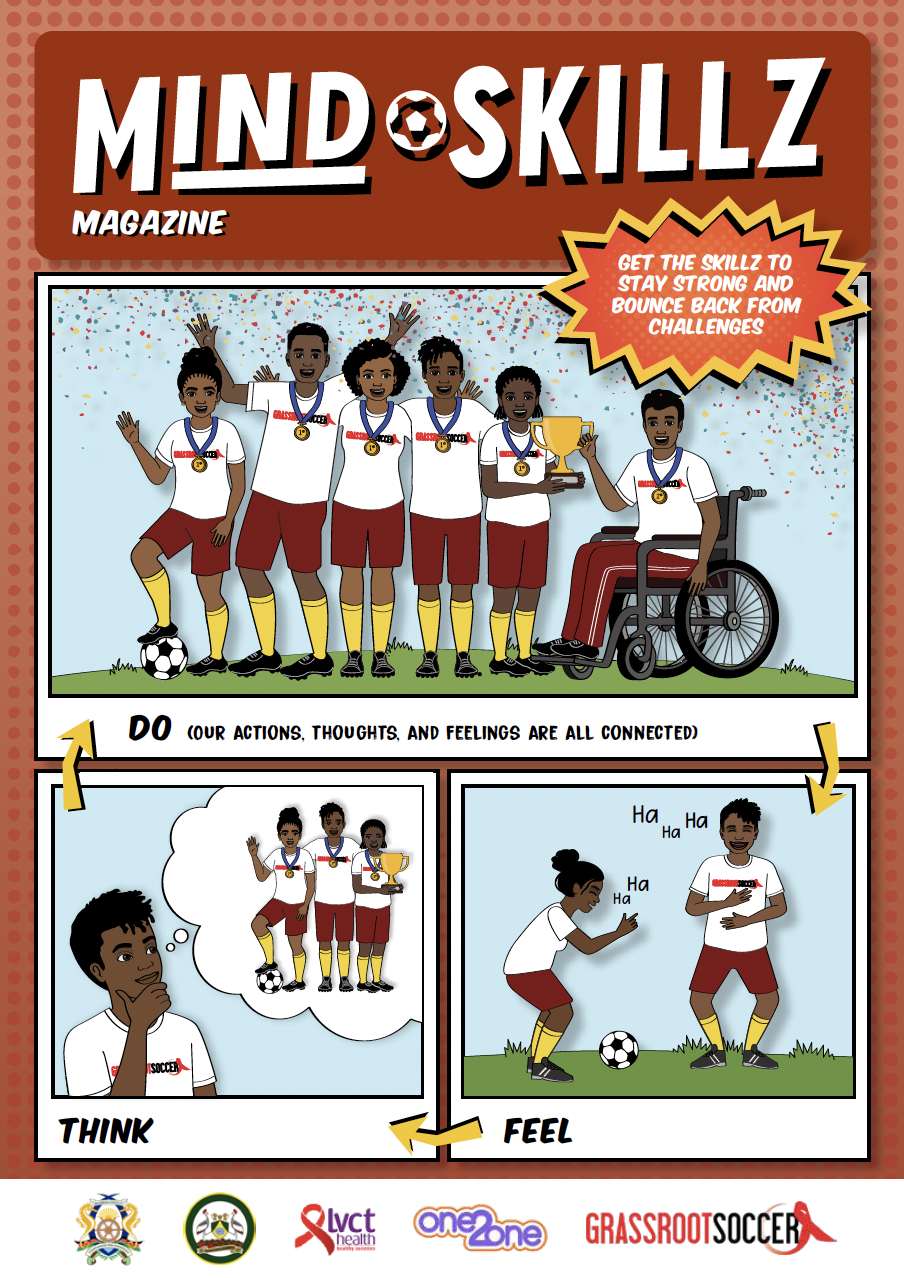
**

**
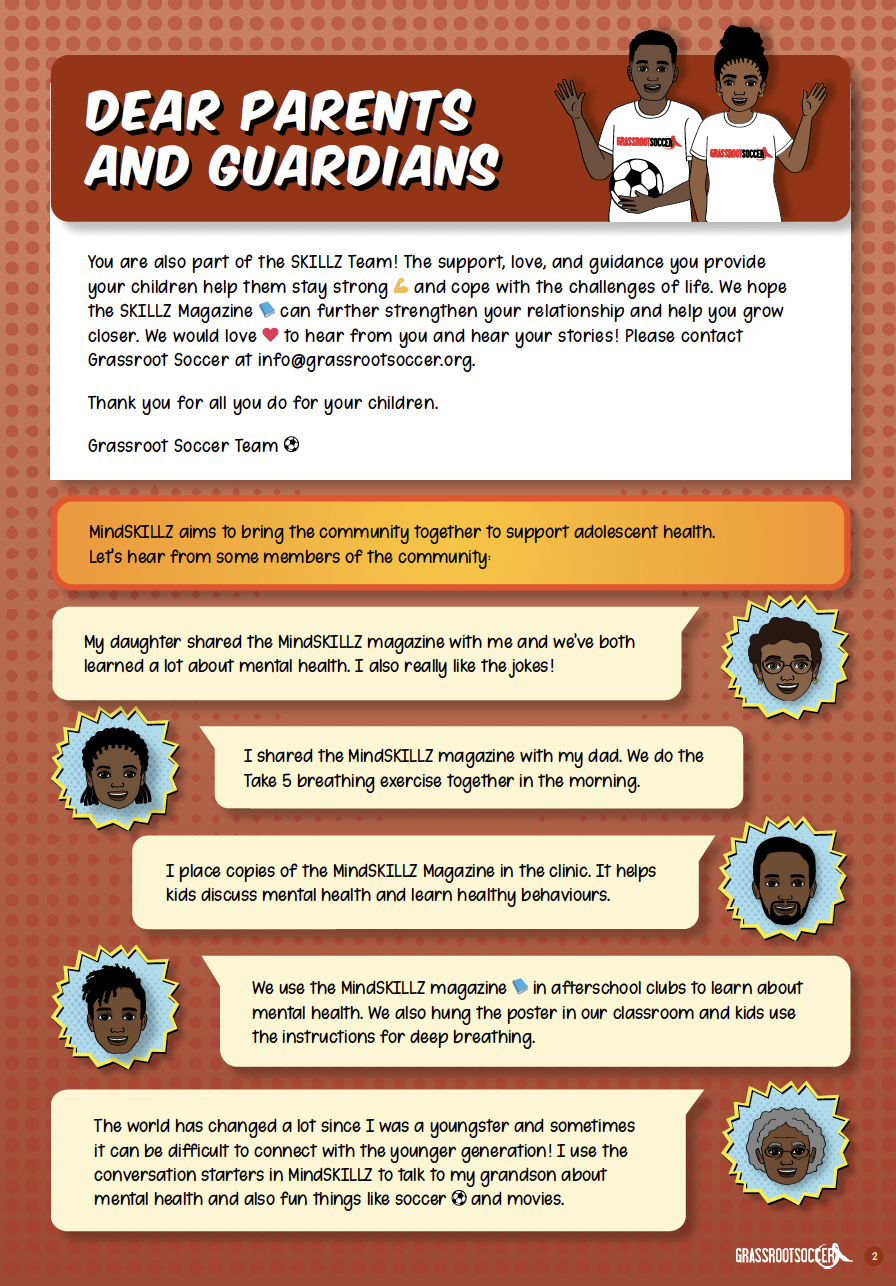
**

**
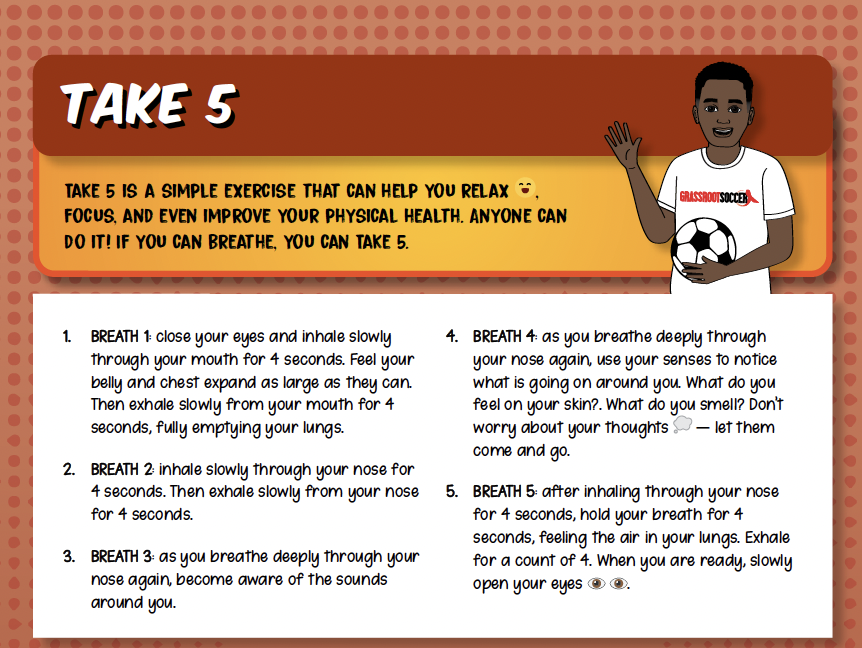
**

**
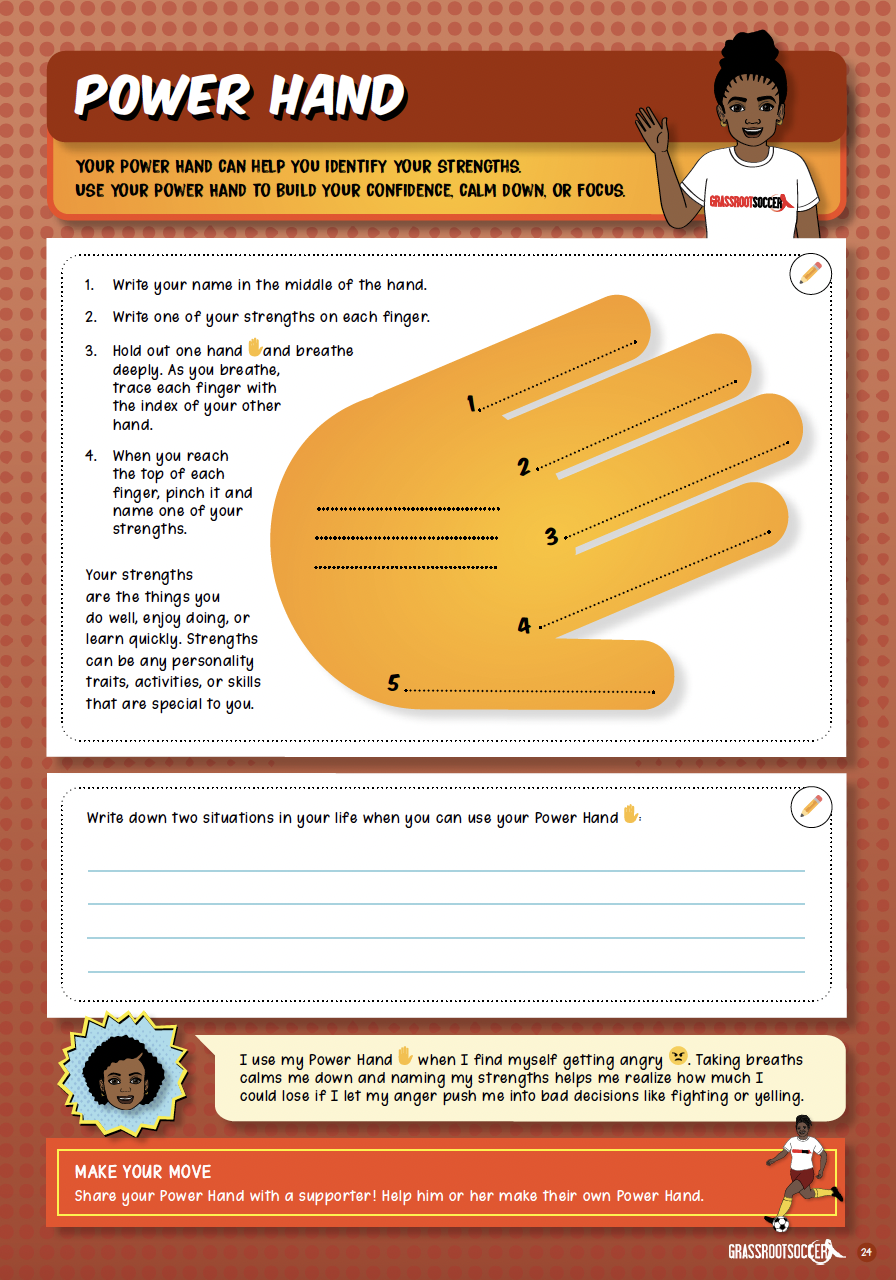
**
